# Dexmedetomidine promotes the progression of hepatocellular carcinoma through hepatic stellate cell activation

**DOI:** 10.1038/s12276-020-0461-6

**Published:** 2020-07-06

**Authors:** Peng Chen, Xiaojun Luo, Guanqi Dai, Yuchuan Jiang, Yue Luo, Shuang Peng, Hao Wang, Penghui Xie, Chen Qu, Wenyu Lin, Jian Hong, Xue Ning, Aimin Li

**Affiliations:** 1 Cancer Center, Integrated Hospital of Traditional Chinese Medicine, Southern Medical University, 510315 Guangzhou, China; 2 Department of Pathophysiology, School of Medicine, Jinan University, 510632 Guangzhou, China; 3 Laboratory of Molecular Medicine, School of Traditional Chinese Medicine, Southern Medical University, 510515 Guangzhou, China; 4 School of Medicine, Jinan University, 510632 Guangzhou, China; 5 Gastrointestinal Unit, Department of Medicine, Massachusetts General Hospital, Harvard Medical School, Boston, MA 02114 USA

**Keywords:** Cancer microenvironment, Cell growth

## Abstract

Dexmedetomidine (DEX) is an anesthetic that is widely used in the clinic, and it has been reported to exhibit paradoxical effects in the progression of multiple solid tumors. In this study, we sought to explore the mechanism by which DEX regulates hepatocellular carcinoma (HCC) progression underlying liver fibrosis. We determined the effects of DEX on tumor progression in an orthotopic HCC mouse model of fibrotic liver. A coculture system and a subcutaneous xenograft model involving coimplantation of mouse hepatoma cells (H22) and primary activated hepatic stellate cells (aHSCs) were used to study the effects of DEX on HCC progression. We found that in the preclinical mouse model of liver fibrosis, DEX treatment significantly shortened median survival time and promoted tumor growth, intrahepatic metastasis and pulmonary metastasis. The DEX receptor (ADRA2A) was mainly expressed in aHSCs but was barely detected in HCC cells. DEX dramatically reinforced HCC malignant behaviors in the presence of aHSCs in both the coculture system and the coimplantation mouse model, but DEX alone exerted no significant effects on the malignancy of HCC. Mechanistically, DEX induced IL-6 secretion from aHSCs and promoted HCC progression via STAT3 activation. Our findings provide evidence that the clinical application of DEX may cause undesirable side effects in HCC patients with liver fibrosis.

## Liver cancer: Common anesthetic can accelerate tumor progression

Researchers warn against using the anesthetic dexmedetomidine (DEX) in liver cancer patients after indications that it promotes tumor growth. Concerns have been raised that certain anesthetics, including DEX, can accelerate the progression of cancerous tumors, but the precise effects of DEX on liver cancer tumors are unclear. Most liver cancers develop in patients who already have fibrosis, a build-up of scarred tissue in the liver. This tissue accumulation stems from the activation of hepatic stellate cells (HSCs) during liver damage. Using human cancer cell lines and mouse models, Aimin Li and Xue Ning at the Southern Medical University, Guangzhou, China and co-workers demonstrated that DEX interacts with HSCs via a receptor protein on their cell surface, further enhancing activation levels. Activated HSCs in turn secrete factors that accelerate tumor growth and invasion.

## Introduction

Hepatocellular carcinoma (HCC) is the second leading cause of cancer-related deaths globally, with an annual incidence of ~850,000 cases^1^. Although chemotherapy, molecular targeted therapy, immunotherapy, photodynamic therapy and transcatheter arterial chemoembolization (TACE) have been applied to HCC^2–5^, surgical resection is considered to be the first-line treatment for HCC patients with well-preserved liver function^6^. Propofol, ropivacaine, lidocaine, and dexmedetomidine (DEX) are commonly used anesthetic drugs in clinics. Emerging evidence has shown that anesthetic drugs play a critical role in HCC progression. Clinically used concentrations of propofol have been reported to promote apoptosis and inhibit the invasion of human HCC cells by regulating related microRNAs^7–9^. Ropivacaine promotes the apoptosis of HCC cells by damaging mitochondria and activating caspase-3^10^. Lidocaine induces profound modifications in the gene expression profiles of tumor cells, including modulating the expression of cell cycle-related genes that result in a cytostatic effect and induction of apoptosis^11^. However, the role of DEX in HCC progression remains to be elucidated.

DEX is an α2A-adrenergic receptor (ADRA2A) agonist^12^ that is widely used in clinics for its unique ability to provide sedation without the risk of respiratory depression and it exhibits opioid- and anesthetic-sparing effects. However, many studies have shown that DEX treatment may cause some unfavorable effects^13–15^, including promoting tumor progression. DEX promotes the progression of breast cancer both in vitro and in vivo via the α2 adrenoceptor, with activation of extracellular signal-related protein kinases^16,17^. In addition, in preclinical models, DEX has been reported to promote the progression of multiple tumors^14,15^. More importantly, in lung cancer patients, a study has suggested that DEX increases the number of Monocytic Meyloid-derived suppressor cells (M-MDSCs) via the α2 adrenoceptor after surgery and promotes tumor metastasis in a mouse model^18^. However, the molecular function of DEX in HCC is poorly understood.

Most HCC develops in the context of liver fibrosis, during which hepatic stellate cells (HSCs) transition from a quiescent to an activated state^19^. Activated HSCs promote HCC progression through the production of a fibrotic stroma and cytokines^20–22^. In breast cancer, α2-adrenergic compounds and DEX promote tumor progression in association with altered collagen structure^23^ and the proliferation of cancer-associated fibroblasts^24^, which are both mainly derived from activated HSCs in HCC. However, the role of DEX in HCC progression, its association with activated HSCs and the underlying mechanisms remain largely unclear.

In the present study, to investigate the impacts of DEX on HCC progression, we used in vitro and in vivo studies, including a syngeneic orthotopic HCC mouse model that recapitulates the key pathological features of a fibrotic liver. Interestingly, our results indicated that DEX had no significant effects on HCC biological behaviors but markedly promoted tumor progression in the presence of activated HSCs. Collectively, our study provides evidence that the clinical application of DEX may cause unfavorable side effects in HCC patients with liver fibrosis.

## Materials and methods

### Cells and drugs

The human HCC cell line Huh7, mouse HCC cell line Hepa1–6, and human hepatic stellate cell line LX-2 were cultured in Dulbecco’s modified Eagle medium (DMEM; Gibco BRL, Grand Island, NY, USA) supplemented with 10% fetal bovine serum (FBS, Gibco BRL), 100 U/ml penicillin, and 100 U/ml streptomycin and incubated at 37 °C under an atmosphere containing 5% CO_2_. Dexmedetomidine (S3075) and Atipamezole (S4650) were purchased from Selleck (Houston, TX, USA).

### Condition medium (CM)

Primary isolated HSCs or LX-2 cells were plated in 35 mm culture dishes at 1 × 10^5^ cells per dish and cultured with DMEM containing 10% FBS overnight. Then, the medium was changed to serum-free DMEM with or without DEX (10 μM). The conditioned medium (CM) was collected after culture for 48 h and filtered through a 0.22 μm membrane. CM was used for proliferation, migration and invasion assays of HCC cells.

### Lentiviral transfection

Short-hairpin RNAs were designed to target ADRA2A (#1: GCACGCTCTTCAAATTCTT; #2: ACCAGAAGTGGTACGTCA; and #3: GCATCAAGGCCATCATCAT) in LX-2 cells. ADRA2A shRNA and control shRNA lentivirus particles were obtained from Genechem (Shanghai, China). Cells were treated with virus-containing supernatant using HiTransG P. After 48 h, 2 μg/ml puromycin was used to select the transfected cells.

### In vitro proliferation, migration, and invasion assays

Cell proliferation was assayed using a CCK-8 kit. Cell migration and invasion assays were performed using Transwell chambers (BD Biosciences) with or without Matrigel (BD Biosciences) according to the manufacturer’s instructions. HCC cells were seeded into the upper chamber in serum-free medium, and CM was added to the lower chamber. After 24 h at 37 °C, the cells remaining in the upper chamber or on the upper membrane were removed with a cotton swab. The cells that had migrated to or invaded to the lower surface of the membrane were stained with a solution containing 0.1% crystal violet and 20% methanol and then photographed with an inverted microscope (Zeiss). The number of positively stained cells was counted using image analysis software (Image-Pro Plus 6.0). At least three independent experiments were performed for each condition.

### Apoptosis assay

Huh7/Hepa1–6 cells were treated with DEX (10 μM) for 48 h, harvested in flow tubes and washed with cool PBS and 1× binding buffer three times. An annexin V-FITC apoptosis detection kit (BD Biosciences) was used to stain the cells, and flow cytometry (FACScan, BD Biosciences, USA) was used to analyze the apoptotic rate of the cells.

### Carboxyfluorescein succinimidyl ester (CFSE) cell proliferation assay

CFSE staining was performed to measure the cell proliferation rate. Huh7 cell suspensions were incubated with 100 μl of 2.5 µM CFSE dye for 10 min at 37 °C in the dark. Stained cells were cultured overnight in 12-well plates in DMEM containing 10% FBS. Then, the medium was changed to CM and cultured for 48 h. A BD FACSCalibur (Spectron Corporation) flow cytometer was used to analyze the samples at a wavelength of 488 nm. A low fluorescent intensity indicated a high proliferation rate.

### Mouse primary hepatic stellate cell isolation

Primary hepatic stellate cells were isolated from male C57BL/6J mice as described previously^25^. In brief, the mice were surgically opened under anesthesia, and the livers were perfused through the inferior vena cava (IVC) with 0.5 mM EGTA, after which the portal vein was cut and the upper IVC was clamped. After 2 min, the livers were perfused with a Pronase (10165921001, Roche) solution for 5 min and then Collagenase-B (11088831001, Roche) solution for 7 min. The livers were removed and minced in a pronase/collagenase/DNAse-I (10104159001, Roche) solution for 24 min and then passed through a 70-μm cell strainer. Liver cells were centrifuged and washed twice in Gey’s balanced salt solution (GBSS) followed by density gradient separation of HSCs using a Histodenz (D2158, Sigma-Aldrich) solution. After centrifugation, HSCs that were located in the interface were collected.

### Isolation of mouse primary hepatocytes, Kupffer cells, and liver sinus epithelial cells

Hepatocytes were isolated by a two-step collagenase perfusion technique as previously described^26^ with modifications. In brief, the mice were surgically opened under anesthesia, and the livers were perfused through the IVC with 0.5 mM EGTA, followed by perfusion with 100 ml of collagenase type IV (Wellington) in Hank’s balanced salt solution (HBSS, containing calcium and magnesium; Gibco). After the liver was digested, it was resected, cut into small pieces and passed through a 70-μm cell strainer. Hepatocytes were separated from nonparenchymal cells (NPCs) by low-speed centrifugation. The NPC fraction was further separated into Kupffer cells (labeled with anti-F4/80 microbeads, 130-110-443, Miltenyi Biotec) and liver sinus epithelial cells (labeled with anti-CD146 microbeads, 130-093-596, Miltenyi Biotec) via a magnetic separation column (Miltenyi Biotech, Auburn, CA) according to the manufacturer’s protocols.

### Hepa1–6 subcutaneous implantation HCC mouse model

Six-week-old male C57/BL mice (Vital River, Beijing, China) received subcutaneous injections of 5 × 10^5^ Hepa1–6 cells. Tumor volumes were measured every 2 days. The following formula was used to calculate the tumor volume: volume = (length × width^2^)/2.

### H22 and pHSC subcutaneous coimplantation HCC mouse model

Primary hepatic stellate cells (pHSCs) were isolated from BALB/c mice (Vital River, Beijing, China) with fibrotic livers. pHSCs (5 × 10^5^) and H22 cells (5 × 10^5^) were resuspended in 200 μl of PBS at a 1:1 ratio. Subcutaneous coimplantation was performed using a 1 ml syringe with a cemented 22-gauge needle. Tumor volumes were measured every 2 days. The following formula was used to calculate tumor volume: volume = (length × width^2^)/2.

### Hepa1–6 orthotopic implantation HCC mouse model with liver fibrosis

Liver fibrosis was induced in 4-week-old male C57BL/6 mice (Vital River, Beijing, China) by CCl_4_ (40% in 100 μl olive oil/mouse, v/v) gavage for 4 weeks. Next, the mice were injected in the subcapsular region of the liver with 25 μl of HCC cell/Matrigel solution (containing 1 × 10^6^ Hepa1–6 cells). Three days after implantation, the mice were treated intraperitoneally with either 10 μg/kg DEX (Selleck Chemicals) or vehicle (*n* = 10 for each group) every day.

### Real-time PCR

Total RNA was isolated according to the standard TRIzol (Takara) method. First, complementary DNA was synthesized from 1 mg of total RNA using PrimeScript RT master mix (Takara). Real-time PCR was performed in an ABI StepOne Real-time Detection System (Life Technologies) using SYBR Green (Takara). The oligonucleotide sequences of the real-time PCR primers are listed in additional file 1.

### Western blotting

Protein samples were collected, and equivalent aliquots of protein were electrophoresed on a 10% sodium dodecyl sulfate/polyacrylamide gel in 1× Tris-glycine buffer, followed by transfer to nitrocellulose membranes and incubation with primary antibodies overnight at 4 °C. Thereafter, the nitrocellulose membranes were incubated with secondary antibodies for 1 h at room temperature. The immunoreactive proteins were detected by an enhanced chemiluminescence substrate; the blot was scanned, and densitometric analysis was performed with ImageJ software.

### Statistical analysis

GraphPad Prism 7.0 software was used for all statistical analyses. Student’s t-test was used to compare values between subgroups, while ANOVA was used to perform comparisons between subgroups with >2 groups. Overall survival (OS) was calculated by Kaplan–Meier survival analysis and log-rank tests. The Pearson correlation test (two-tailed) was used to calculate the correlation coefficient. D’Agostino and Pearson normality tests were used to determine the normality of distributions. The variance within each group of data was compared using the *F*-test (two groups) or Brown–Forsythe test (more than two groups). The data are expressed as the mean ± standard deviation (SD) of at least three biological replicates. Statistical significance was set at *P* < 0.05.

## Results

### DEX promotes the progression of tumors in a mouse HCC orthotopic model with liver fibrosis

**Fig. 1:**
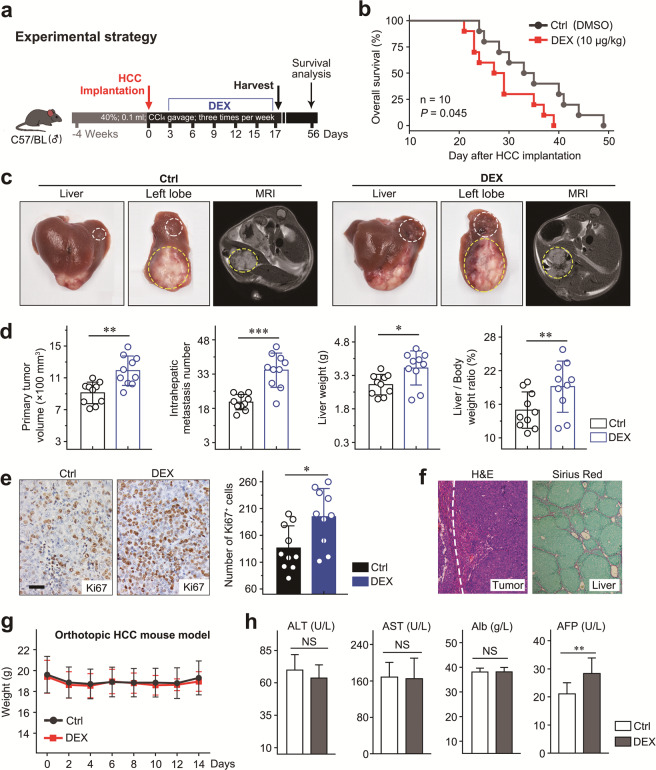
DEX promoted tumor growth and metastasis in an orthotopic HCC mouse model. **a** Experimental design for exploring the effects of DEX on tumor progression in an orthotopic HCC mouse model of liver fibrosis. Mice were subjected to MRI (magnetic resonance imaging) before harvesting at day 14 after HCC implantation. **b** Kaplan–Meier survival analysis of HCC mice that received vehicle or DEX (10 μg/kg; *n* = 10/group). **c** Representative image of mouse gross liver, left lobe, and MRI; scale bars, 500 mm. **d** Statistical analysis of the primary tumor volume, intrahepatic metastasis number, liver weight and liver/body weight ratio. **e** Representative image of mouse HCC tissue stained with Ki67 (left panel) and statistical analysis of their IHC score (right panel); scale bars, 100 μm. **f** Representative H&E image of mouse HCC tissue (left panel) and representative Sirius red image of mouse paratumor tissue (right panel). **g** DEX had no significant effect on body weight. **h** Serum levels of ALT, AST, Alb, and AFP. Data are presented as the mean ± SD. NS = not significant. *P < 0.05; **P < 0.01; ***P < 0.001.

To investigate the effects of DEX on HCC progression, we established an orthotopic mouse model with liver fibrosis induced by carbon tetrachloride (CCl_4_), which recapitulated the key pathological features of HCC. DEX (10 μg/kg) or vehicle control was administered and mice were subjected to magnetic resonance imaging and tumor measurement at the time of sacrifice (Fig. 1a). Survival analysis revealed that while all mice died within 49 days in our aggressive fibrosis-associated HCC model (Fig. 1b), mice treated with DEX showed shorter survival times than their vehicle controls (median survival: 28 vs. 34 days, *P* = 0.045; Fig. 1b). Notably, compared with vehicle treatment, DEX treatment markedly promoted the progression of HCC, which was accompanied by significant increases in tumor volume and intrahepatic metastasis numbers (mean diameter: 11.90 ± 1.75 vs. 9.12 ± 1.30 mm, *P* < 0.01; *P* < 0.01, respectively; Fig. 1c, d). Furthermore, we observed that mice treated with DEX had increased liver weights and liver/body weight ratios (*P* < 0.05 and *P* < 0.01, respectively).

To better understand the mechanisms leading to increased HCC growth, we evaluated tumor proliferation using Ki67 staining. We found that HCC cell proliferation was increased by 58% in the DEX treatment group (Fig. 1e). Furthermore, H&E and Sirius red staining were used to confirm the formation and fibrotic microenvironment of HCC, respectively (Fig. 1f). In addition, DEX treatment was well tolerated, and neither liver function impairment nor body weight loss was observed, but DEX induced increased secretion of AFP (Fig. 1g, h).

### ADRA2A, the DEX receptor, is expressed in nontumor fibrotic liver tissue and is mainly located in activated HSCs but barely detected in HCC

**Fig. 2:**
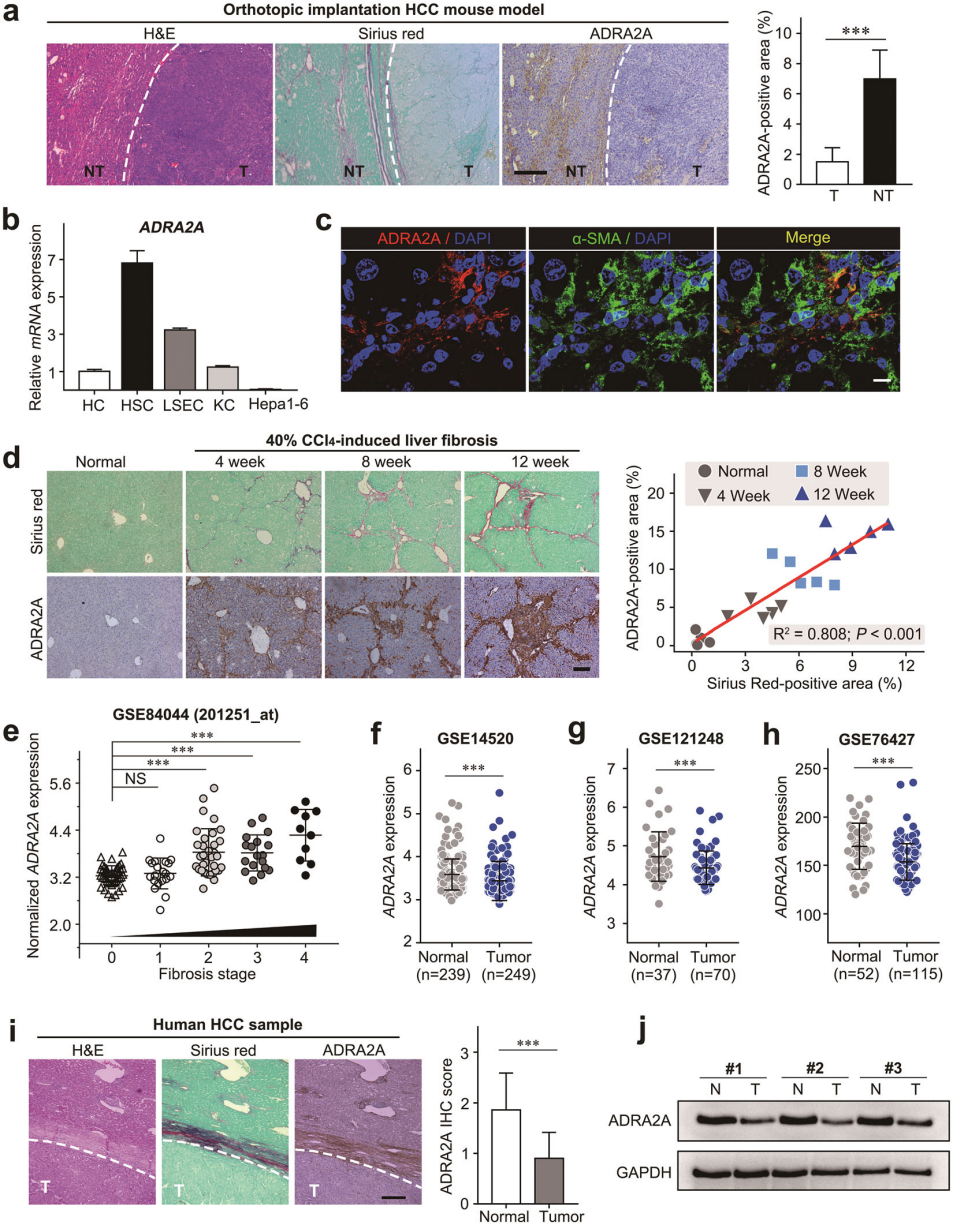
ADRA2A was mainly expressed in aHSCs but was barely detected in HCC cells. **a** H&E, Sirius red, and ADRA2A IHC were performed in mouse orthotopic HCC tissue. Representative images (left panel) and statistical analysis of ADRA2A IHC (right panel) are shown; T tumor, NT nontumor; scale bars, 100 μm. **b** The ADRA2A mRNA level in mouse primary hepatic cells (HC hepatocyte, KC Kupffer cell, LSEC, liver sinus endothelial cell, HSC hepatic stellate cell) and mouse HCC cell (Hepa1–6). **c** Double immunofluorescence staining of ADRA2A and α-SMA in mouse fibrotic tissue. Scale bars, 10 µm. **d** Representative Sirius red image and ADRA2A IHC image of mouse normal liver and progressively fibrotic liver (left panel). Correlation between ADRA2A IHC staining and Sirius red staining (right panel); scale bars, 100 μm. **e** The publicly available database from the National Cancer for Biotechnology Information Gene Expression Omnibus (GSE84044) was used to explore the role of ADRA2A in liver fibrosis/cirrhosis. **f**, **g** *ADRA2A* expression was investigated in public datasets: GSE14520, GSE121248, and GSE76427. **i** H&E, Sirius red, and ADRA2A IHC were performed in HCC patient tissue, and the representative images are shown; T tumor; scale bars, 100 μm (left panel), the statistical analysis of ADRA2A expression in HCC tissues and their adjacent normal tissues (right panel). **j** ADRA2A expression was detected by western blotting. Data are presented as the mean ± SD. ***P < 0.001.

To explore the possible functions by which DEX promotes HCC progression, we first detected the expression of ADRA2A, a well-reported DEX receptor, in serial sections of orthotopic implantation mouse HCC tissues. We found that ADRA2A was mainly expressed in nontumor tissue and colocalized to areas of collagen deposition but was barely present in mouse HCC tissue (Fig. 2a). To further detect the location of ADRA2A, we isolated primary hepatic cells (HCs, hepatocytes; KCs, Kupffer cells; LSECs, liver sinus epithelial cells; HSCs, hepatic stellate cells) from a CCl_4_-induced primary mouse HCC model. Consistently, ADRA2A was predominantly expressed in activated HSCs (Fig. 2b) and colocalized with α-SMA (an activated HSC marker) (Fig. 2c). More than 80% of HCC develops from underlying liver fibrosis/cirrhosis and stromal activation, which are key elements of a microenvironment that is conducive to tumorigenesis. We found that the expression of ADRA2A gradually increased with the development of CCl_4_-induced liver fibrosis and had a positive correlation with the degree of collagen deposition (*R*^2^ = 0.808, *P* < 0.001) (Fig. 2d). The publicly available database from the National Cancer for Biotechnology Information Gene Expression Omnibus (GEO, GSE84044) was used to explore the role of ADRA2A in liver fibrosis/cirrhosis. Interestingly, we found that the ADRA2A mRNA was significantly increased in human fibrotic livers in association with fibrosis progression (Fig. 2e). These data indicated that DEX can exert its effects on HCC by regulating the functions of activated HSCs.

Liver fibrosis is a key factor that contributes to hepatocarcinogenesis; therefore, we detected the expression of ADRA2A in HCC. Using the publicly available databases GSE14520 (which includes gene expression profiles from 239 normal liver and 249 HCC tissues), GSE121248 (which includes gene expression profiles from 37 normal liver and 70 HCC tissues) and GSE76427 (which includes gene expression profiles from 52 normal liver and 115 HCC tissues), we found that *ADRA2A* mRNA expression was downregulated in HCC tissue compared normal controls (Fig. 2f–h). Furthermore, to verify the results from the public database, we detected the expression of ADRA2A via immunochemistry in another 50 pairs of HCC patient samples. The results showed that 10 of 50 (20%) HCC samples and 45 of 50 (90%) corresponding nontumor tissues were positively stained (Fig. 2i). Consistently, in three pairs of fresh HCC samples, we found that by western blotting ADRA2A expression was downregulated in HCC tissues compared to matched adjacent nontumor tissues (Fig. 2j).

### DEX has no significant effects on the pathogenesis of HCC in vitro and in vivo

**Fig. 3:**
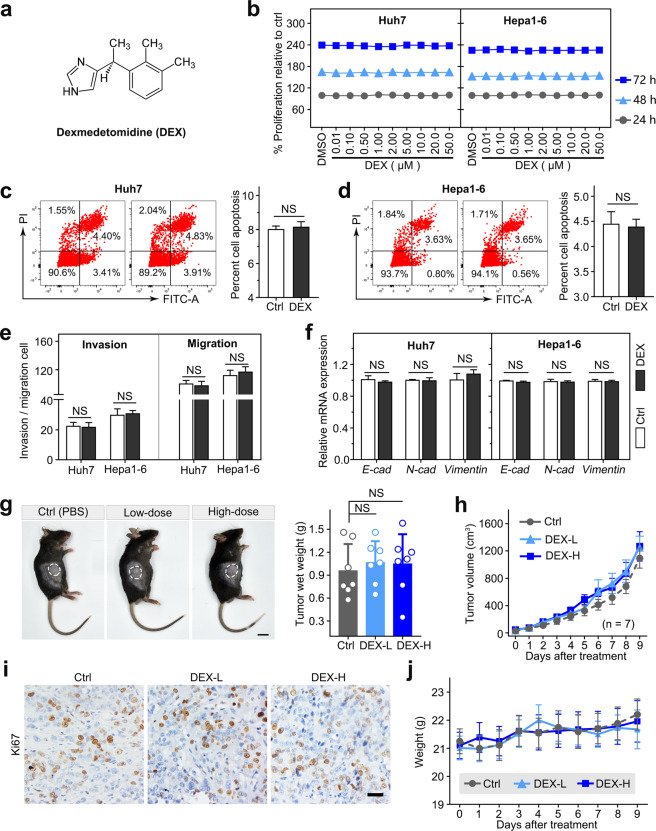
DEX did not exert significant effects on the biological behaviors of HCC in vitro or in vivo. **a** The molecular formula of DEX. **b** Cell viability of Huh7 and Hepa1–6 cells treated with DEX for 24, 48, and 72 h. **c**, **d** Huh7/Hepa1–6 cells were stained with FITC-conjugated annexin V and PI after DEX treatment and monitored using flow cytometry. **e** The invasion/migration ability of Huh7/Hepa1–6 cells in the presence of DEX (10 μM). **f** The mRNA level of EMT-related biomarkers in Huh7/Hepa1–6 cells treated with DEX (10 μM). **g** Representative gross image of mice from the indicated groups (left panel). Histogram analysis of the weight of tumors harvested at the end point (right panel). **h** The tumor volume [(length × width^2^)/2] after treatment of mice with vehicle or DEX (L, low: 5 μg/kg; H, high: 10 μg/kg). Data are presented as the mean ± SEM. **i** Ki67 IHC staining was performed in HCC tissue. **j** Mouse body weight during DEX treatment. Data are presented as the mean ± SD. NS not significant.

It has been reported that DEX, a common sedative used in the clinic, promotes the proliferation and invasion of multiple tumors. Here, we investigated the effects of DEX on hepatoma cells. The molecular formula of DEX is shown in Fig. 3a. We found that various concentrations of DEX (0.01–50 μM) for 24, 48, and 72 h had no significant effects on the viability of Huh7/Hepa1–6 cells (Fig. 3b). Similarly, no significant difference in cell apoptosis was observed between the DEX treatment and vehicle control treatment (Fig. 3c, d). In addition, DEX treatment had no marked effects on the malignancy of Huh7/Hepa1–6 cells, as indicated by Transwell migration and invasion assays, and it did not significantly alter the expression of EMT-related genes, including *E-cadherin*, *N-cadherin*, and *vimentin*, in these two cell lines (Fig. 3e, f). Next, to determine the effect of DEX on the progression of HCC in vivo, a subcutaneous HCC mouse model that was xenografted with Hepa1–6 cells was utilized. Compared to control treatment, DEX treatment had no significant effects on tumor growth at low or high doses (Fig. 3g–i). Furthermore, no obvious body weight change was observed during DEX treatment in any of the three groups (Fig. 3j). Collectively, these data suggested that DEX treatment has no significant effects on the biological behaviors of HCC.

### DEX promotes HSC activation via ADRA2A

**Fig. 4:**
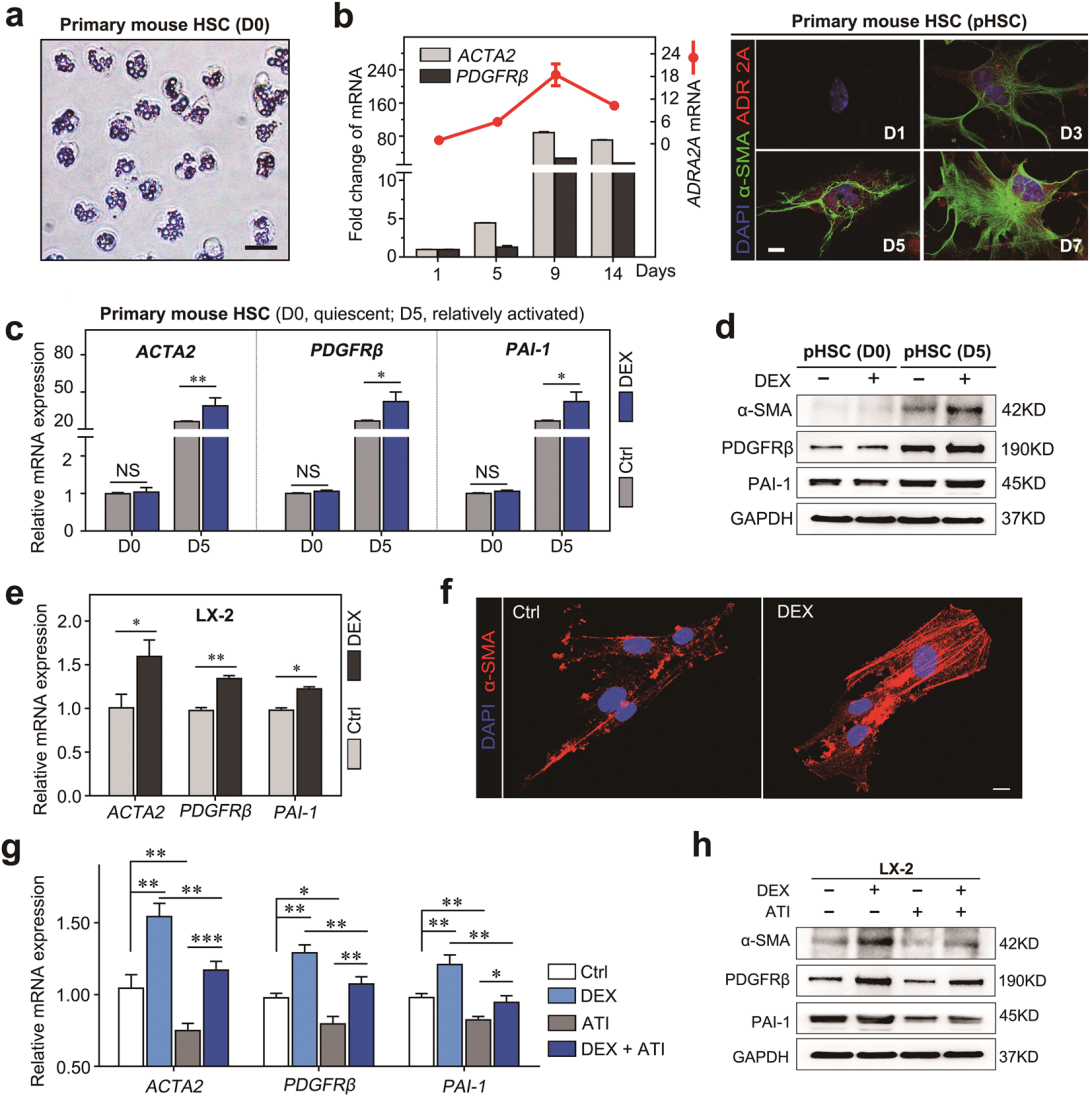
DEX enhanced the activation of aHSCs but had no significant effect on quiescent HSCs. **a** Representative image of freshly isolated HSCs from C57/BL mice. Merging of the retinoid fluorescence image with the phase-contrast image shows complete overlap of the retinoid signal with characteristic lipid droplets. **b** The left panel shows fold changes in *ADRA2A*, *ACTA2*, and *PDGFRβ* mRNA expression in primary HSCs isolated from normal mouse livers cultured for the indicated time intervals. The right panel shows gradually increasing protein levels of ADRA2A and α-SMA in primary HSCs isolated from normal mouse liver cultured for the indicated time intervals. **c** *ACTA2*, *PDGFRβ*, and *PAI-1* mRNA level changes in D0 cells (quiescent HSCs) and D5 cells (partially aHSCs) cultured with DEX (10 μM). **d** ACTA2, PDGFRβ, and PAI-1 protein level changes in D0 cells (quiescent HSCs) and D5 cells (relatively aHSCs) cultured with DEX (10 μM). **e** *ACTA2*, *PDGFRβ*, and *PAI-1* mRNA level changes in LX-2 cells treated with DEX. **f** Immunofluorescence staining of α-SMA in LX-2 cells treated with DEX (10 μM). **g** *ACTA2*, *PDGFRβ*, and *PAI-1* mRNA level changes in LX-2 cells treated with DEX, ATI (atipamezole), or the combination. **h** Changes in ACTA2, PDGFRβ, and PAI-1 protein levels in LX-2 cells treated with DEX, ATI, or the combination. Data are presented as the mean ± SD. *P < 0.05; **P < 0.01.

It has been reported that primary HSCs gradually become activated within 2 weeks of being plated on plastic dishes. Thus, to elucidate the role of ADRA2A, which is a well-reported DEX receptor, in HSC activation, we isolated primary HSCs from mouse livers as previously described and obtained high purity HSCs as indicated by retinoid fluorescence (Fig. 4a). We examined *ADRA2A* expression along with that of HSC activation markers (*ACTA2* and *PDGFRβ*) in cultured primary mouse HSCs at different stages of differentiation (from day 1 to 14). We found that the mRNA level of *ADRA2A* gradually increased during HSC activation, as indicated by the upregulation of *ACTA2* and *PDGFRβ* (Fig. 4b). ADRA2A protein expression was detected by immunofluorescence at an early stage of HSC activation, confirming that ADRA2A expression increased in parallel with that of the HSC activation marker (α-SMA) (Fig. 4b).

DEX is a fast-acting and robust α2A-adrenergic receptor (ADRA2A) agonist. Interestingly, we found that DEX had no significant effects on the activation of quiescent HSCs that did not express ADRA2A, but DEX promoted the expression of various HSC activation genes (*ACTA2*, *PDGFRβ*, and *PAI-1*) in activated HSCs (Fig. 4c, d). These data indicated that DEX may exert its effect on HSC activation via ADRA2A. To confirm this result, we treated the human activated HSC cell line LX-2 with DEX (10 μM) and found that DEX treatment promoted the activation of LX-2 and the expression of HSC activation genes, including *ACTA2*, *PDGFRβ*, and *PAI-1* (Fig. 4e, f). Conversely, atipamezole, an ADRA2A antagonist, inhibited the expression of HSC activation genes, including *ACTA2*, *PDGFRβ*, and *PAI-1*, and neutralized the effects of DEX on HSC activation (Fig. 4g, f). Taken together, our findings strongly suggest that DEX exerts its effects on HSCs via ADRA2A.

### DEX promotes the proliferation and metastasis of HCC in an HSC-dependent manner

**Fig. 5:**
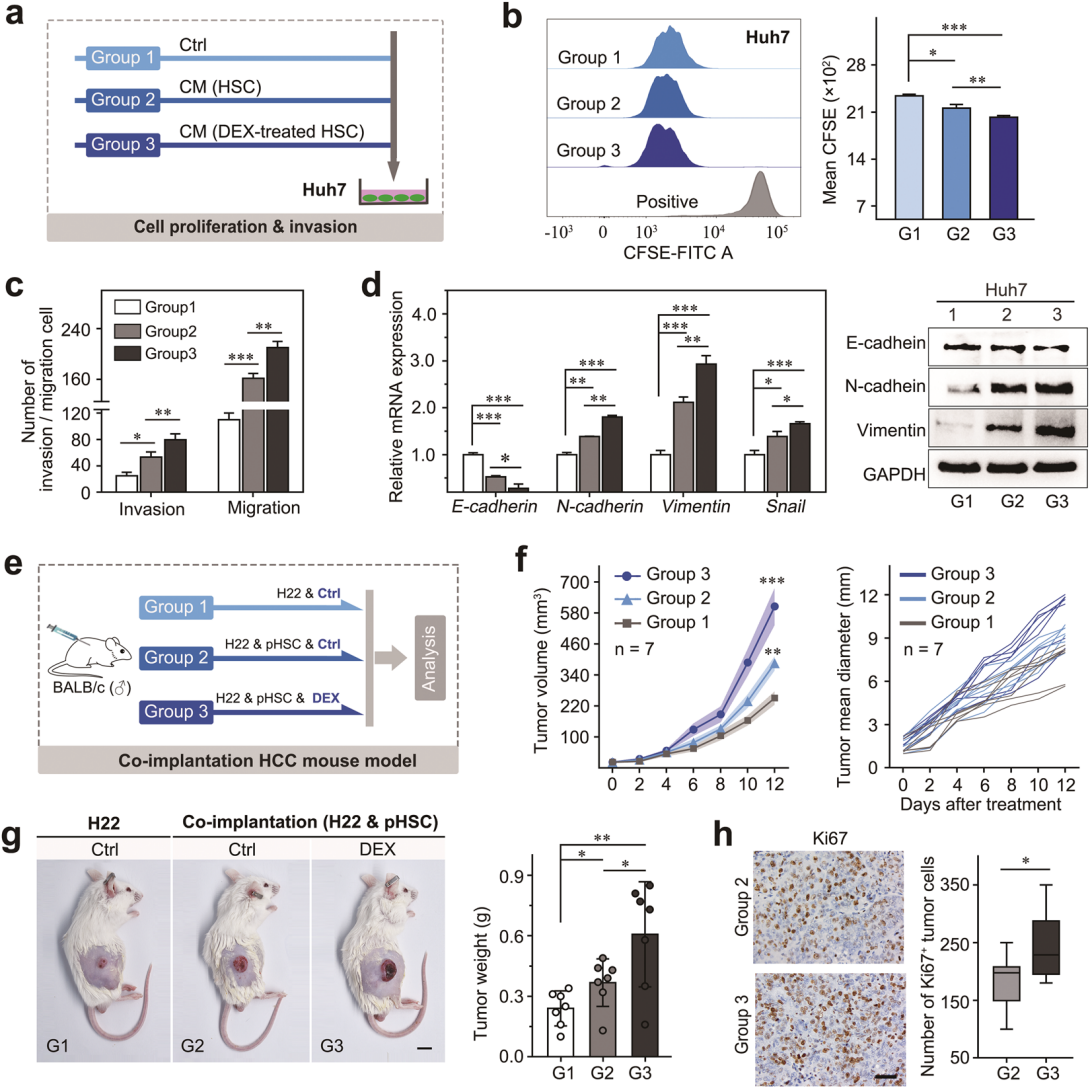
DEX promoted the proliferation and metastasis of HCC in the presence of aHSCs. **a** Experimental design for investigating the effects of DEX on HCC cell proliferation and metastasis in the presence of HSCs. **b** The effect of DEX (10 μM)-pretreated HSC CM on Huh7 cell proliferation indicated by CFSE assay. **c** The invasion/migration ability of Huh7 cells in the presence of DEX-pretreated HSC CM. **d** The mRNA level (left panel) and protein level (right panel) of the indicated EMT-related biomarkers in Huh7 cells treated with DEX were investigated by RT-PCR and western blotting. **e** Experimental design to investigate the effects of DEX on HCC growth in H22 (mouse HCC cells) and primary HSC coimplantation models. **f** Measurement of the tumor volume [(length × width^2^)/2] (left panel) and tumor mean diameter (right panel) in H22 and primary HSC coimplantation mice that were treated with vehicle or DEX (10 μg/kg); data are presented as the mean ± SEM. **g** Representative image of a mouse from the indicated group (left panel) and statistical analysis of tumor weight at the end point (right panel). **h** Representative image of Ki67 IHC of mouse HCC tissue (left panel) and statistical analysis of Ki67 IHC results (right panel) are shown. Data are presented as the mean ± SD. *P < 0.05; **P < 0.01.

Activated HSCs can promote the malignancy of HCC in a paracrine manner. To elucidate whether DEX treatment promotes HCC progression via HSC activation, we treated hepatoma cells with CM from activated HSCs in the presence of DEX (Fig. 5a). Huh7 cells had increased proliferation when cocultured with LX-2 cells, and the growth-promoting effect was further enhanced when LX-2 cells were pretreated with DEX (Fig. 5b). In addition, compared to the control or to CM from activated HSCs, CM from DEX-pretreated LX-2 cells increased the migration and invasive abilities of Huh7 cells (Fig. 5c). Similarly, our study revealed that CM from activated HSCs significantly upregulated the expression of EMT-related genes in Huh7 cells, including E-cadherin, N-cadherin, vimentin, Snail, and DEX treatment further exacerbated this effect (Fig. 5d).

To further confirm this finding in vivo, we established a subcutaneous HCC model by engrafting mice with both H22 cells (mouse hepatoma cells) and primary activated HSCs (isolated from BALB/c mice with fibrotic livers) (Fig. 5e). We found that the addition of activated HSCs supported the growth of HCC, and DEX treatment further promoted tumor growth (Fig. 5f, g). In addition, Ki67 immunohistochemical staining revealed that DEX treatment supported robust proliferation abilities in the cancer cells in the presence of activated HSCs (Fig. 5h). These data strongly indicate that DEX promotes the progression of HCC in an activated HSC-dependent manner.

### DEX-mediated HCC progression depends on ADRA2A, which induces IL-6 secretion in activated HSCs

**Fig. 6:**
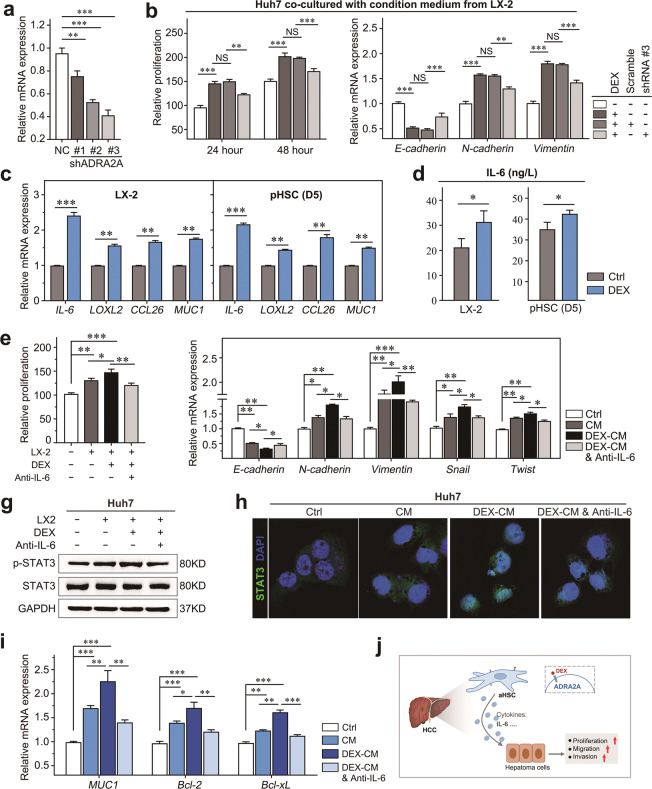
DEX induced tumor-promoting cytokine and chemokine secretion from aHSCs to promote HCC proliferation, metastasis, and STAT3 activation. **a** The expression of *ADRA2A* in LX-2 cells was detected by RT-PCR. **b** The proliferation ratio (left panel) and EMT-related biomarker expression (right panel) in Huh7 cells measured by CCK-8 and RT-PCR, respectively. **c** Changes in cytokine and chemokine (*IL-6*, *LOXL2*, *CCL26*, *MUC1*) mRNA levels in LX-2 or primary aHSCs treated with DEX. **d** The concentration of IL-6 in the culture supernatant was measured by ELISA after 48 h of DEX (10 μM) treatment. **e** The proliferation ratio of Huh7 cells was detected by CCK-8. **f** The mRNA levels of the indicated EMT-related biomarkers in Huh7 cells were investigated by RT-PCR. **g** Huh7 cells were inoculated in monoculture or in HSC CM or exposed to 100 ng/ml IL-6 neutralizing antibody. The protein levels of STAT3 and p-STAT3 in Huh7 cells were detected after 48 h inoculation. **h** Representative images of STAT3 changes in Huh7 cells from the indicated groups were investigated by confocal microscopy. **i** Changes in the mRNA levels of STAT3 downstream molecules (*MUC1*, *Bcl2*, *Bcl-xL*) in Huh7 cells were investigated by RT-PCR. **j** Graphical abstract illustrating the key concept that DEX induces IL-6 secretion from activated HSCs, which promotes the proliferation, migration, and invasion of HCC cells. Data are presented as the mean ± SD. *P < 0.05; **P < 0.01; ***P < 0.001.

To investigate whether DEX promotes the progression of HCC mainly through its receptor (ADRA2A) on HSCs, ADRA2A expression was knocked down using shRNA (Fig. 6a), and Huh7 cells were cultured in the CM of LX-2 cells treated with/without DEX. We found that DEX significantly enhanced the malignancy of Huh7 cells, whereas ADRA2A knockdown partly impaired the promoting effects of DEX on Huh7 cell proliferation and EMT marker expression (Fig. 6b).

Chemokines and cytokines play a critical role in the promotion of HCC^27,28^. To investigate the mechanisms underlying the growth-facilitating effect of DEX that is mediated by activated HSCs, we first detected the expression of major protumor chemokines and cytokines in LX-2 cells and primary mouse HSCs by qPCR, including *IL-6*, *LOXL2*, *CCL26*, and *MUC1* (Fig. 6c). We focused on IL-6, which has been reported to be produced by HSCs in the HCC microenvironment, where it facilitates tumor progression. Moreover, compared to control treatment, DEX treatment elevated the concentration of IL-6 in the supernatant of both LX-2 cells and primary mouse HSCs (Fig. 6d). The growth-promoting effect of DEX-pretreated CM on Huh7 cells was abolished by the presence of anti-IL-6 neutralizing antibody, and this effect was accompanied by downregulation of EMT-related gene expression (Fig. 6e, f). STAT3 pathway activation is known to be a vital factor in HCC progression. We found that CM from activated HSCs promoted the activation of STAT3; in addition, increased STAT3 activation was observed in Huh7 cells that were exposed to DEX-pretreated CM, while IL-6 neutralizing antibody partially abolished this effect (Fig. 6g, h). In addition, we examined the downstream targets of STAT3 by detecting the expression of *MUC1*, *Bcl2*, and *Bal-xL*, which further confirmed the above findings (Fig. 6i). In summary, DEX induced the secretion of tumor-promoting cytokines (IL-6, etc.) from activated HSCs via ADRA2A and promoted the progression of HCC (Fig. 6j).

## Discussion

DEX is a fast-acting and robust α2A-adrenergic receptor (ADRA2A) agonist that is commonly used as an anesthetic in the clinic. Many studies have indicated that DEX exerts paradoxical effects on the progression of multiple solid tumors. In this study, we established an orthotopic implantation HCC mouse model with liver fibrosis to investigate the role of DEX in tumor progression. Our data demonstrated that DEX had no significant effects on the proliferation or invasion of HCC cells in vitro and in vivo but promoted the progression of HCC in the presence of activated HSCs.

Recently, an increasing number of studies have suggested that DEX promotes the progression of multiple solid tumors in mouse models, partly by increasing the proliferation and metastatic activity of cancer cells^14,15^. Consistently, DEX has also been reported to dose-dependently restore lidocaine-impaired proliferation of PC12 cells by decreasing the expression of the tumor suppressor protein p21 and increasing the expression of cyclin D1 and CDK1^29^. In addition, DEX increases the number of CD11b + CD33 + HLA-DR-CD14 + M-MDSCs in lung cancer patients after thoracotomy and promotes tumor metastasis by increasing the production of VEGF^18^. However, in an ovarian cancer rat model, DEX exerted a protective effect that enhanced immune surveillance by inhibiting the p38/MAPK/NF-κB signaling pathway^30^. Interestingly, our data indicated that DEX had no significant effects on the biological behaviors of HCC in vitro or in a subcutaneous implantation mouse model, but DEX promoted the progression of HCC in the presence of activated HSCs. Thus, these data indicate that the application of DEX in the clinic, especially in HCC surgery, should be avoided and reexamined. Importantly, further clinical trials and basic studies are indeed needed to clarify the paradoxical roles of DEX in tumor progression and the related mechanisms.

Most HCC develops in the setting of liver fibrosis, which is characterized by the accumulation of excess ECM^31^. The ECM is predominantly derived from activated HSCs, which account for 5% to 8% of the cell population in the liver and are the key effectors of liver fibrosis^32–34^. Thus, in this study, we not only utilized an orthotopic HCC implantation mouse model with liver fibrosis to investigate the role of DEX in tumor progression but also explored the correlation between ADRA2A expression and the ECM in mouse liver fibrotic tissues. Some studies have demonstrated that DEX promotes breast cancer progression by altering collagen structure and enhancing fibroblast proliferation^23,24^, both of which are derived from activated HSCs in HCC. Consistently, our study showed that DEX promoted the malignancy of HCC by enhancing HSC activation. Activated HSCs secrete several humoral factors and accelerate tumor proliferation, invasion and angiogenesis^32–34^. IL-6, CCL2, VEGFA, and MUC1, which are derived from activated HSCs, have been reported to promote HCC progression by facilitating vascular angiogenesis and tissue remodeling. IL-6 has been reported to be produced by HSCs in the HCC microenvironment, where it facilitates the progression of HCC^32^. In the experiments, we found that DEX induced IL-6 secretion by activated HSCs and promoted HCC cell proliferation and invasion through STAT3 activation. Although our data indicated that IL-6 was related to DEX-induced HCC progression associated with activated HSCs, other molecules might also be involved in this process.

G-protein coupled receptors (GPCRs) represent the largest family of membrane proteins in the human genome^35^. Adrenoreceptors, which belong to the GPCR family, play an important role in regulating body homeostasis in health and disease^36^. Three genes, namely ADRA2A, ADRA2B, and ADRA2C, have been identified in several species that encode the α2 adrenoreceptor^37^. ADRA2A has been reported to be expressed in normal fibroblasts but is barely detected in cancer-associated fibroblasts. In breast cancer, α2-adrenergic agonists, including DEX, enhance the proliferation of fibroblasts and promote tumor progression^24^. Similarly, we found that ADRA2A was barely expressed in quiescent HSCs but was upregulated in activated HSCs, which are the main source of fibroblasts or myofibroblasts in the liver^38–40^. DEX enhanced the activity of activated HSCs, whereas atipamezole, an ADRA2A antagonist, rescued this effect and inhibited the activation of HSCs. These data indicated that ADRA2A is a plausible target for liver fibrosis treatment. However, we did not examine the expression of the other two subtypes of α2 adrenoreceptors in HSCs and liver tissue.

In summary, we investigated the effects of DEX on the progression of HCC in vitro and in vivo. Interestingly, we found that DEX had no significant effects on the malignancy of HCC but promoted the growth and metastasis of tumors in the presence of activated HSCs. Mechanistically, DEX induced the secretion of IL-6 from activated HSCs via ADRA2A, which promoted STAT3 activation and HCC progression. Our study indicates that the clinical application of DEX may cause unfavorable side effects in HCC patients with liver fibrosis and calls for prospective clinical trials to evaluate the impact of perioperative dexmedetomidine use on the outcomes of HCC patients.

## Supplementary information

Supplemental materials: Dexmedetomidine promotes the progression of hepatocellular carcinoma through hepatic stellate cell activation
