# Supplementary material for: Dexmedetomidine promotes the progression of hepatocellular carcinoma through hepatic stellate cell activation
Source: Exp Mol Med. 2020 Jul 6;52(7):1062–74. doi: 10.1038/s12276-020-0461-6 (PMC8080602; doi:10.1038/s12276-020-0461-6)
Supplement: Supplementary file 1 — Supplemental materials: Dexmedetomidine promotes the progression of hepatocellular carcinoma through hepatic stellate cell activation [file 12276_2020_461_MOESM1_ESM.doc]

Supplemental materials: Dexmedetomidine Promotes the Progression of Hepatocellular Carcinoma through Hepatic Stellate Cell Activation

RT-qPCR Primers Used in The Study

| **Target Name** | **Species** | **Forward Primer**  **Reverse Primer** | |
| --- | --- | --- | --- |
| *E-cadherin* | Human | 5’ - TCGACACCCGATTCAAAGTGG - 3’ | |
|  |  | 5’ - TTCCAGAAACGGAGGCCTGAT - 3’ | |
| *N-cadherin* | Human | 5’ - GCGCGTGAAGGTTTGCCAGTG - 3’ | |
|  |  | 5’ - CCGGCGTTTCATCCATACCACAA - 3’ | |
| *Vimentin* | Human | 5’ - TGGCCGACGCCATCAACACC - 3’ | |
|  |  | 5’ - CACCTCGACGCGGGCTTTGT - 3’ | |
| *Snail* | Human | 5’ - AAGGATCTCCAGGCTCGAAAG - 3’ | |
|  |  | 5’ - GCTTCGGATGTGCATCTTGA - 3’ | |
| *Twist* | Human | 5’ - GGAGTCCGCAGTCTTACGAG - 3’ | |
|  |  | 5’ - TCTGGAGGACCTGGTAGAGG - 3’ | |
| *ACTA2* | Human | 5’ - CGTGGCTATTCCTTCGTTAC - 3’ | |
|  |  | 5’ - TGCCAGCAGACTCCATCC - 3’ | |
| *PDGFRβ* | Human | 5’ - GCCCTTATGTCGGAGCTGAAGA - 3’ | |
|  |  | 5’ - GTTGCGGTGCAGGTAGTCCA - 3’ | |
| *PAI-1* | Human | 5’ - AGTGGACTTTTCAGAGGTGGA - 3’ | |
|  |  | 5’ - GCCGTTGAAGTAGAGGGCATT - 3’ | |
| *IL6* | Human | 5’ - TTCTCCACAAGCGCCTTC - 3’ | |
|  |  | 5’ - AGCAGGCAACACCAGGAG - 3’ | |
| *CCL26* | Human | 5’ - CTGGACCTGGGTGCGAAGC - 3’ | |
|  |  | 5’ - TGGATGGGTACAGACTTTCTTGCC - 3’ | |
| *MUC1* | Human | 5’ - GCAGCAGCCTCTCTTACACAAAC - 3’ | |
|  |  | 5’ - AGAACCTGAGTGGAGTGGAATGG - 3’ | |
| *LOXL2* | Human | 5’ - AGTGACTCATCTTCCTGTTGTTCC - 3’ | |
|  |  | 5’ - GACCCTGGTTATAGCACCGTTG - 3’ | |
| *GAPDH* | Human | 5’ - GGCATGGACTGTGGTCATGAG - 3’ | |
|  |  | 5’ - GGCATGGACTGTGGTCATGAG - 3’ | |
| *Bcl-xl* | Human | 5’ - CCCAGAAAGGATACAGCTGG - 3’ | |
|  |  | 5’ - GCGATCCGACTCACCAATAC - 3’ | |
| *Bcl-2* | Human | 5’ - TTCTTTGAGTTCGGTGGGGTC - 3’ | |
|  |  | 5’ - TGCATATTTGTTTGGGGCAGG - 3’ | |
| *ACTA2* | mouse | 5’ - TGGCCGACGCCATCAACACC - 3’ | |
|  |  | 5’ - CACCTCGACGCGGGCTTTGT - 3’ | |
| *PDGFRβ* | mouse | 5’ - AGCCAGAAGTAGCGAGAAGC - 3’ | |
|  |  | 5’ - GGCAGTATTCCGTGATGATG - 3’ | |
| *PAI-1* | mouse | 5’ - GACACCCTCAGCATGTTCAT - 3’ | |
|  |  | 5’ - AGGGTTGCACTAAACATGTCAG - 3’ | |
| **Target Name** | **Species** | **Forward Primer**  **Reverse Primer** | |
| *ADRA2A* | mouse | | 5’ - CTTTTGCACGTCGTCCATAGT - 3’ |
|  |  | | 5’ - CGGTGACAATGATGGCCTTGA - 3’ |
| *IL-6* | mouse | | 5’ - GGCGGATCGGATGTTGTGAT - 3’ |
|  |  | | 5’ - GGACCCCAGACAATCGGTTG - 3’ |
| *MUC1* | mouse | | 5’ - CGTCAGGCTCAGCTATCATTC - 3’ |
|  |  | | 5’ - GGGTATTGACTTGGCACTGAA - 3’ |
| *LOXL2* | mouse | | 5’ - CGATGTGGTCAAGATCCAGGT - 3’ |
|  |  | | 5’ - TGGCCTCTACATAGCCCACTT - 3’ |
| *CCL26* | mouse | | 5’ - TTCTTCGATTTGGGTCTCCTTG - 3’ |
|  |  | | 5’ - GTGCAGCTCTTGTCGGTGAA - 3’ |
